# Self-Treatment of Posterior Canal Benign Paroxysmal Positional Vertigo: A Preliminary Study

**DOI:** 10.3389/fmed.2021.654637

**Published:** 2021-04-29

**Authors:** Zhuangqin Gan, Shiling Zhou, Hui Yang, Feng He, Dong Wei, Ya Bai, Yuanyuan Wang, Yingxia Wang, Wei Fu, Junliang Han

**Affiliations:** ^1^Department of Neurology, Qionghai People's Hospital, Qionghai, China; ^2^Department of Neurology, Xijing Hospital, Fourth Military Medical University, Xi'an, China; ^3^Department of Geriatrics, Xijing Hospital, Fourth Military Medical University, Xi'an, China

**Keywords:** benign paroxysmal positional vertigo, Epley maneuver, modified, residual symptoms, self-treatment, semicircular canal

## Abstract

**Objectives:** The purpose of this study is to investigate a modified Epley maneuver for self-treatment of posterior canal benign paroxysmal positional vertigo (PC-BPPV).

**Methods:** The study recruited 155 patients with PC-BPPV. All patients were randomized into the Epley maneuver group (*n* = 77) and modified Epley maneuver group (*n* = 78). We analyzed the resolution rate (1 day and 1 week), residual symptoms after the maneuver, and adverse effects.

**Results:** It was found that the modified Epley maneuver group had a higher resolution rate than that of the Epley maneuver group in the treatment of PC-BPPV after 1 day of the initial maneuver (*p* < 0.05). However, there was no difference in resolution rate between the Epley maneuver group and the modified Epley maneuver group in resolution rate after 1 week of the initial maneuver (*p* > 0.05). The modified Epley maneuver group had fewer residual symptoms than that of the Epley maneuver group 1 week after treatment of PC-BPPV (*p* < 0.05). Significant improvements were also observed in average DHI scores in patients who underwent the modified Epley maneuver compared to the Epley maneuver (*p* < 0.05). There was no significant difference in adverse effects between the two groups (*p* > 0.05).

**Conclusions:** The modified Epley maneuver has a satisfactory therapeutic efficacy with less residual symptoms and could be recommended as a self-treatment for patients with PC-BPPV.

## Introduction

Benign paroxysmal positional vertigo (BPPV) is the common cause of peripheral vertigo. It is usually caused by otoconia that are dislodged from the otolith macula beds and become trapped in the semicircular canal (1). Approximately 60-90% of BPPV involves the posterior canal (PC-BPPV) (2). PC-BPPV is usually diagnosed by the means of the Dix–Hallpike test, which is considered positive when it triggers vertigo symptoms as well as torsional and vertical nystagmus (3). Treatment of PC-BPPV relies on a canalith repositioning maneuver, and the most common approach is the Epley maneuver (4). In a retrospective study, 84% of patients obtained symptomatic control of PC-BPPV after three Epley maneuvers (5), which means repeated Epley maneuvers are required for some patients until PC-BPPV resolves completely. PC-BPPV also has a high recurrence rate (6, 7) and frequent recurrences render patients with PC-BPPV dependent on costly and time-consuming medical care. Thus, the self-treatment approach is a desirable option for patients with PC-BPPV. In this study, a modified Epley maneuver for self-treatment at home was introduced and its therapeutic efficacy evaluated and compared to the conventional Epley maneuver.

## Patients and Methods

### Patients

Between October 2019 and October 2020, 155 consecutive patients with a diagnosis of unilateral PC-BPPV were recruited at the neurotology unit of our clinic. All patients underwent routine nervous system examination and laboratory examinations. The diagnosis of PC-BPPV was performed using the diagnostic criteria established by the Bárány Society in 2015 (3), which included a history of recurrent transient positional vertigo and induced positional nystagmus by the Dix–Hallpike test. The nystagmus is a combination of torsional nystagmus with the upper pole of the eyes beating toward the lower ear combined with vertical nystagmus beating upward (toward the forehead) typically lasting <1 min. We used Frenzel to observe nystagmus to eliminate visual fixation.

The exclusion criteria for this study were: (1) horizontal canal and multicanal BPPV; (2) other vestibular disorders, such as vestibular neuritis, vestibular migraine; (3) central nervous system disorders; and (4) declined to participate in this study.

All subjects provided written informed consent to participate in this study and the study was approved by the Qionghai People's Hospital Ethics Committee.

### Treatment Maneuvers: Epley Maneuver and Modified Epley Maneuver

All patients with PC-BPPV were randomly assigned to the Epley maneuver group (*n* = 77, 35 males and 42 females, mean age: 57.85 ± 14.22 years) and modified Epley maneuver group (*n* = 78, 31 males and 47 females, mean age: 57.26 ± 13.12 years). The demographic and clinical characteristics of patients with PC-BPPV are shown in Table 1. For the Epley maneuver, the patient was held in the head hanging position for 30 s (4), and then, the head was turned 90° toward the unaffected side. This position was maintained for 30 s before turning the head another 90°, so the head was nearly in the face-down position. The patient was then brought to the sitting-up position (4). After reexamination with the Dix-Hallpike test 30 min to 1 h later, when the patient still showed vertigo or positioning nystagmus, the patient received the Epley maneuver again. We performed a maximum of two reexaminations. Then patients were arranged for everyday follow up for 1 week after the initial maneuver.

**Table 1:** Demographic and clinical characteristics of patients with posterior canal benign paroxysmal positional vertigo.

	**Epley maneuver (*n* = 77)**	**Modified Epley maneuver (*n* = 78)**	***p-*value**
Age (mean ± SD), years	57.85 ± 14.22	57.26 ± 13.12	0.78^a^
Sex (female/male)	42/35	47/31	0.47^b^
Affected side (right/left)	45/32	42/36	0.82^b^
Duration of symptom (mean ± SD), days	6.94 ± 7.02	6.01 ± 7.10	0.41^a^
Resolution rate, *n* (%)			
1 day follow up	58 (75.32)	70 (89.74)	0.01^b^
1 week follow up	71 (92.21)	75 (96.15)	0.32^c^
Residual symptoms, *n* (%)	45 (58.44)	25 (32.05)	<0.01^b^

a *The p-values were computed using t test*,

b *Chi-square test, or*

c *Fisher's exact test*.

**Figure 1:**
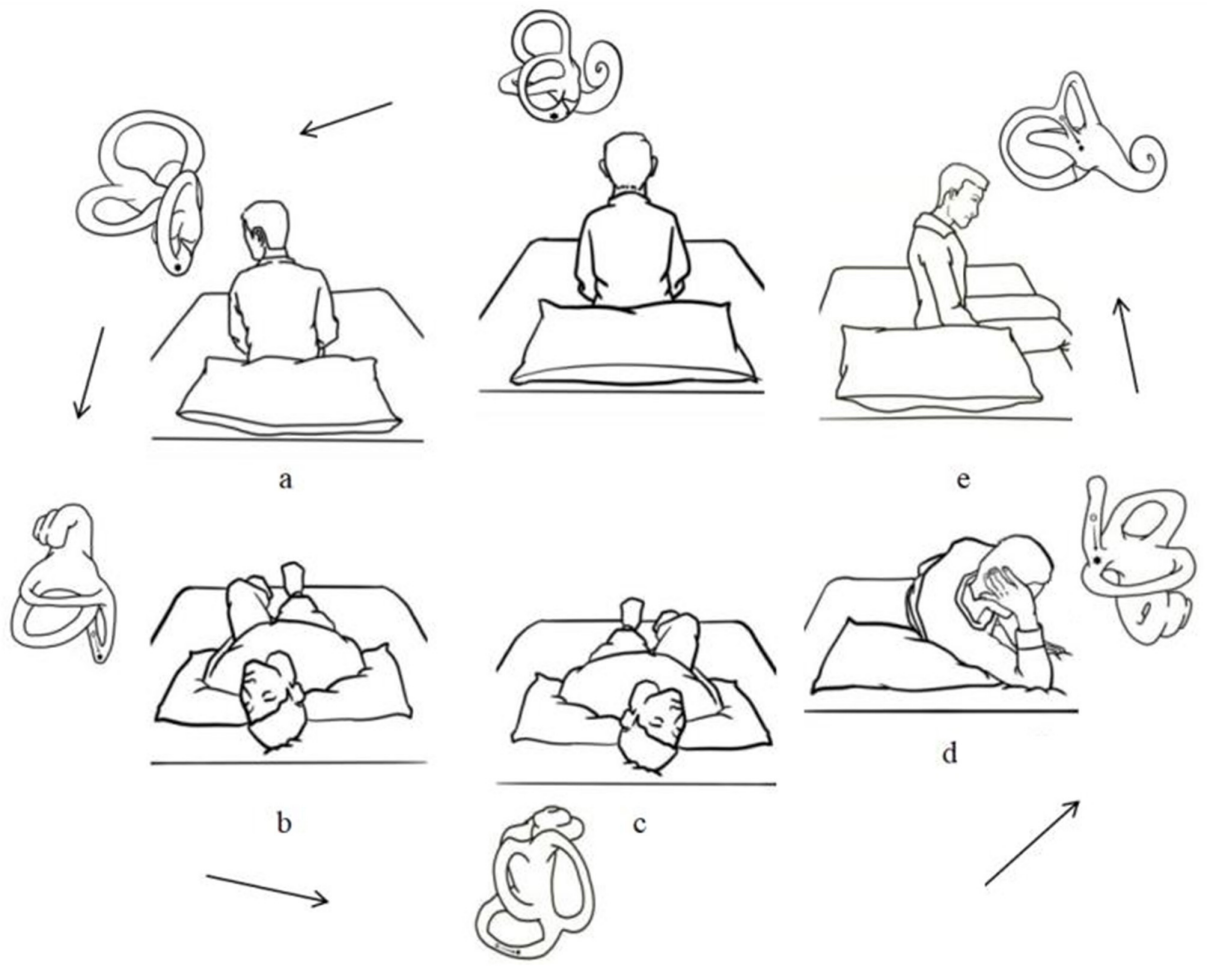
Illustration of a modified Epley maneuver for self-treatment of posterior canal benign paroxysmal positional vertigo. **(A)** The patient's head was turned 45° to the affected side in the sitting position. **(B)** The patient was laid on the bed and head deflexibility (during which the head hangs at about 20°) was achieved by supporting the patient's shoulder with a pillow for 30 s. **(C)** The patient was held turning the head 90° toward the unaffected side for 30 s. **(D)** Turning the head and body another 90° and the body was in a side-lying position toward the unaffected side for 30 s. **(E)** The patient was brought to the sitting up position and head inclined forward 30°.

For the modified Epley maneuver, all patients received an illustrated instruction of a specific maneuver. Patients perform it by themselves and the sequence of head and body movements was explained. The patient's head was turned 45° to the affected side in the sitting position. Then the patient was laid on the bed and head deflexibility (during which the head hangs at about 20°) was achieved by supporting the patient's shoulder with a pillow for 30 s. The patient was held turning the head 90° toward the unaffected side. This position was maintained for 30 s before turning the head and body another 90°, so the body was in a side-lying position toward the unaffected side. Meanwhile, the patient's head was held by the hands. This position was maintained for 30 s before the patient was brought to the sitting up position and head inclined forward 30° (Figure 1). Patients were asked to repeat self-treatment three times daily at home and were arranged for everyday follow up for 1 week after the initial maneuver.

### Evaluation of Treatment Outcome

All patients were classified as resolution (the absence of both vertigo and nystagmus) and ineffective (the non-remission or exacerbation of vertigo or nystagmus). The resolution rate was calculated by the formula as follows: $the\;resolution\;rate\;=\frac{\text{the number of resolution patients}}{\text{the number of total patients}}$. Furthermore, the residual symptoms after the maneuver and adverse effects were recorded. Residual symptoms imply a non-specific sensation of unsteadiness, lightheadedness, disorientation, fogginess, or drowsiness (8). In addition, all patients need to complete the Chinese version of the dizziness handicap inventory (DHI-P, DHI-E, and DHI-F) questionnaire before and 1 week after the initial maneuver.

### Statistical Analysis

Statistical analysis included Student's *t*-test for continuous variables, the chi-square test, and Fisher's exact test to compare proportions. The cumulative effect of each maneuver was determined by Kaplan-Meier survival analysis and was compared among the groups using the log-rank test. Statistical analyses were done using SPSS software (version19, SPSS Inc., Chicago, IL, USA) with a statistical significance at *p* < 0.05.

## Results

One day after the initial maneuver, 58 (75.32%) patients reported resolution and 19 (24.68%) patients reported ineffective in the Epley maneuver group. In the modified Epley maneuver group, 70 (89.74%) patients reported resolution and 8 (10.26%) patients reported ineffective. The resolution rate was significantly higher in the modified Epley maneuver group compared with the Epley maneuver group (*p* < 0.05, Table 1).

**Figure 2:**
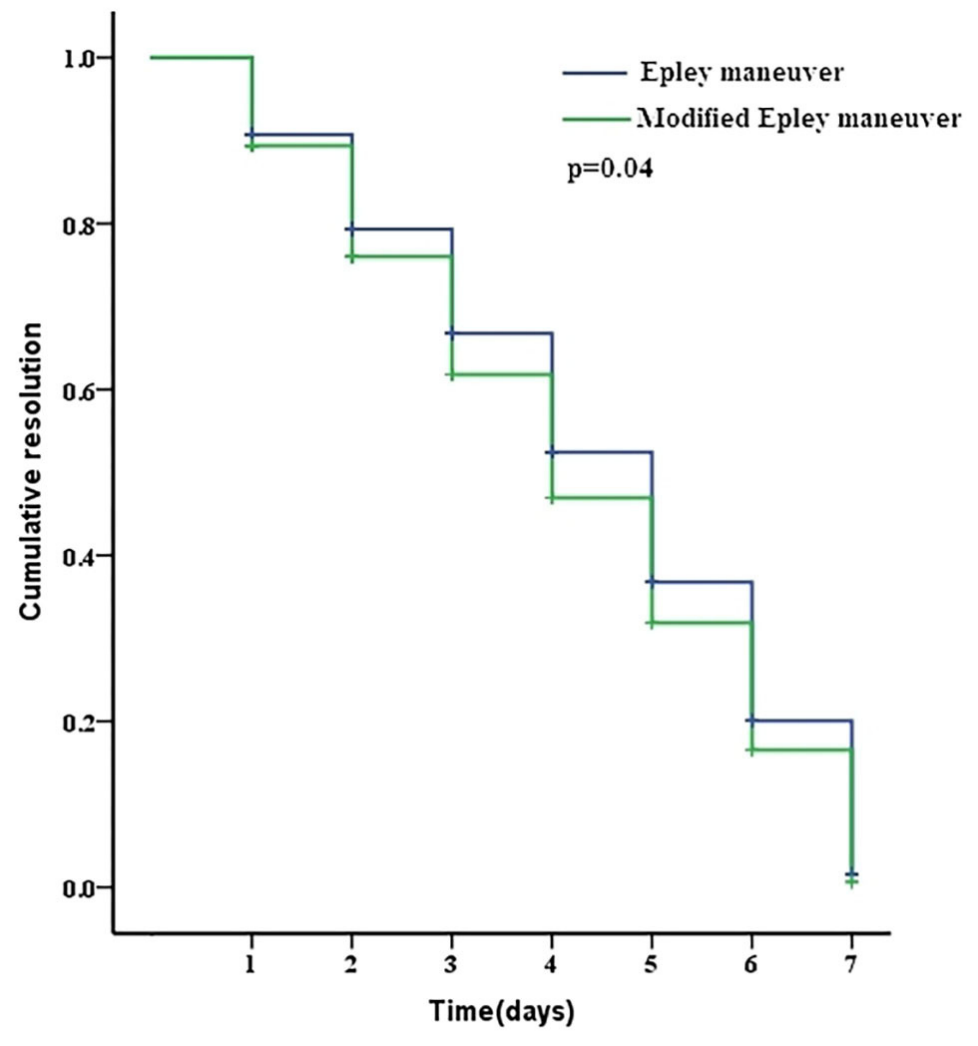
The Kaplan-Meier survival curve with a log-rank test for cumulative therapeutic effects on patients with PC-BPPV after Epley maneuver and modified Epley maneuver treatment.

One week after the initial maneuver, 71 (92.21%) patients reported resolution, and 6 (7.79%) patients reported ineffective in the Epley maneuver group. In the modified Epley maneuver group 75 (96.15%) patients reported resolution and 3 (3.85%) patients reported ineffective. No difference was observed in the resolution rate between the modified Epley maneuver group and Epley maneuver group (*p* > 0.05, Table 1). The Kaplan-Meier survival curve with a log-rank test for cumulative therapeutic effects at 1 week follow up showed a better outcome with modified Epley maneuver (*p* < 0.05, Figure 2).

45 (58.44%) patients had residual symptoms after the Epley maneuver at 1 week. 25 (32.05%) patients had residual symptoms after modified Epley maneuver at 1 week. The modified Epley maneuver group had fewer residual symptoms than the Epley maneuver group (*p* < 0.05, Table 1).

Table 2 shows the average DHI scores (DHI-P, DHI-E, and DHI-F) in the week before and 1 week after the Epley maneuver and modified Epley maneuver. The average DHI scores before the Epley maneuver and modified Epley maneuver were 45.81 ± 21.36 and 45.97 ± 19.30. The average DHI scores 1 week after the Epley maneuver and modified Epley maneuver were 22.62 ± 22.91 and 13.97 ± 21.92. There were significant improvements in the average DHI scores in patients who underwent the Epley maneuver and modified Epley maneuver (*p* < 0.05). Furthermore, significant improvements were observed in average DHI scores (DHI-P, DHI-E, and DHI-F) in patients who underwent the modified Epley maneuver compared to those in the Epley maneuver (*p* < 0.05).

**Table 2:** DHI scores in initial and after 1 week follow up.

		**Epley maneuver (*n* =77)**	**Modified Epley maneuver (*n* = 78)**	***p-*value**
Initial DHI score	Functional	19.24 ± 9.19	19.10 ± 8.40	0.91^a^
	Emotional	10.46 ± 8.28	10.17 ± 7.62	0.82^a^
	Physical	15.89 ± 5.23	16.56 ± 4.83	0.41^a^
	Total	45.81 ± 21.36	45.97 ± 19.30	0.96^a^
1 week follow up DHI score	Functional	9.81 ± 9.64	6.33 ± 9.29	0.02^a^
	Emotional	7.48 ± 7.57	4.41 ± 6.77	<0.01^a^
	Physical	5.24 ± 6.35	3.02 ± 5.87	0.02^a^
	Total	22.62 ± 22.91	13.97 ± 21.92	0.01^a^

*DHI, dizziness handicap inventory*.

a *The p-values were computed using t-test*.

Epley maneuver and modified Epley maneuver have not found serious adverse effects. 21 (27.27%) patients reported nausea, vomiting, and muscle soreness in the Epley maneuver. 16 (20.51%) patients reported nausea, vomiting, and muscle soreness in the modified Epley maneuver. There was no significant difference between the two groups (*p* > 0.05).

## Discussion

This study found that self-treatment with modified Epley maneuver is more effective than Epley maneuver in treating PC-BPPV after 1 day follow up. The modified Epley maneuver had less residual symptoms after 1 week follow up. However, there was no difference in therapeutic efficacy between the modified Epley and Epley maneuvers after 1 week follow up.

Some studies reported that Epley maneuver had success rates in treating PC-BPPV, ranging from 79.6% after 1 day follow up to nearly 100% after 1 week follow up (9). Our study yielded similar results since the response rates of 75.32 and 92.20% after 1 day and 1 week follow up were observed, which could be explained by the number of maneuvers. Some studies showed that the canalith repositioning maneuver in treating PC-BPPV should be repeatedly applied (5, 10). Thus, patients with PC-BPPV needed to be treated several times, which is inconvenient.

In our study, we applied a modified Epley maneuver for the self-treatment of PC-BPPV, which achieved satisfactory efficacy. The resolution rates at the 1 day and 1 week follow up intervals were 89.74 and 96.15%, respectively, which was higher than that of the Epley maneuver.

In previous studies, Radtke et al. found that the success rate was 95% for self-treatment with modified Epley maneuver in PC-BPPV at follow up evaluation after 1 week, which was similar to our result (11). However, in the study by Radtke et al. the modified Epley maneuver was not compared with the Epley maneuver. Our study confirmed that the resolution rate with the modified Epley maneuver was superior to the Epley maneuver at follow up evaluation after 1 day. Unsuccessful treatment with modified Epley maneuver was related to incorrect maneuver execution or insufficient follow up time (12).

Despite the high success rate of the canalith repositioning maneuver in treating BPPV, some patients still report a non-specific sensation of unsteadiness, lightheadedness, disorientation, fogginess, or drowsiness named residual dizziness (8). The overall prevalence of residual dizziness ranges from 31 to 61% which does not seem to be related to the involvement of the semicircular canal (13, 14). The duration of residual dizziness can range from days to several weeks (15, 16).

The prevalence of residual symptoms in our study was approximately half of the patients (58.44%) in the Epley maneuver group and one third of the patients (32.05%) in the modified Epley maneuver group, which was similar to previous reports. It was found that the prevalence of residual symptoms had a significant difference when comparing the two groups. This difference might be explained by remaining otoconial debris due to incomplete canalith repositioning and insufficient central adaptation after successes between two maneuvers (17, 18). The DHI score was used to assess patients with PC-BPPV in dizziness-related physical impairments, activity limitations, and restrictions after 1 week follow up and it was found that three subdomains of DHI (functional, emotional, and physical) were alleviated and much more frequent in the modified Epley maneuver. Thus, this self-treatment maneuver is suitable for treating patients with PC-BPPV. In our study, both maneuvers were well-tolerated by the patients and no serious adverse effects were found.

The primary limitation of this study was the number of patients. A future study with a large sample needs to further confirm the results.

## Conclusion

Most patients with PC-BPPV require repeated canalith repositioning maneuver. This study confirmed that the modified Epley maneuver for self-treatment has a satisfactory therapeutic efficacy with less residual symptoms, which could be a viable tool for patients with frequent recurrences rendering them independent from costly and time-consuming medical care. Thus, it could be recommended as a self-treatment approach for patients with PC-BPPV and also be considered as a complementary treatment, especially for patients who fail to respond to a single Epley maneuver.

## Data Availability Statement

The original contributions presented in the study are included in the article/Supplementary Material, further inquiries can be directed to the corresponding author/s.

## Ethics Statement

The studies involving human participants were reviewed and approved by Qionghai People's Hospital Ethics Committee. The patients/participants provided their written informed consent to participate in this study.

## Author Contributions

ZG, SZ, and HY designed the experiment, collected data, and wrote the article. FH, DW, YB, YuW, and YiW analyzed data and prepared figures. WF and JH guided the study. All authors contributed to the article and approved the submitted version.

## Conflict of Interest

The authors declare that the research was conducted in the absence of any commercial or financial relationships that could be construed as a potential conflict of interest.
